# Oncogenic extracellular vesicles in brain tumor progression

**DOI:** 10.3389/fphys.2012.00294

**Published:** 2012-07-24

**Authors:** Esterina D’Asti, Delphine Garnier, Tae H. Lee, Laura Montermini, Brian Meehan, Janusz Rak

**Affiliations:** Pediatrics, Cancer and Angiogenesis Laboratory, RI MUHC, Montreal Children’s Hospital, McGill UniversityMontreal, QC, Canada

**Keywords:** extracellular vesicles, exosomes, oncogenes, cancer, brain

## Abstract

The brain is a frequent site of neoplastic growth, including both primary and metastatic tumors. The clinical intractability of many brain tumors and their distinct biology are implicitly linked to the unique microenvironment of the central nervous system (CNS) and cellular interactions within. Among the most intriguing forms of cellular interactions is that mediated by membrane-derived extracellular vesicles (EVs). Their biogenesis (vesiculation) and uptake by recipient cells serves as a unique mechanism of intercellular trafficking of complex biological messages including the exchange of molecules that cannot be released through classical secretory pathways, or that are prone to extracellular degradation. Tumor cells produce EVs containing molecular effectors of several cancer-related processes such as growth, invasion, drug resistance, angiogenesis, and coagulopathy. Notably, tumor-derived EVs (oncosomes) also contain oncogenic proteins, transcripts, DNA, and microRNA (miR). Uptake of this material may change properties of the recipient cells and impact the tumor microenvironment. Examples of transformation-related molecules found in the cargo of tumor-derived EVs include the oncogenic epidermal growth factor receptor (EGFRvIII), tumor suppressors (PTEN), and oncomirs (miR-520g). It is postulated that EVs circulating in blood or cerebrospinal fluid (CSF) of brain tumor patients may be used to decipher molecular features (mutations) of the underlying malignancy, reflect responses to therapy, or molecular subtypes of primary brain tumors [e.g., glioma or medulloblastoma (MB)]. It is possible that metastases to the brain may also emit EVs with clinically relevant oncogenic signatures. Thus, EVs emerge as a novel and functionally important vehicle of intercellular communication that can mediate multiple biological effects. In addition, they provide a unique platform to develop molecular biomarkers in brain malignancies.

## Introduction– intercellular communication in complex biological systems

The fascination with biological identity tends to overshadow the inherent interconnectedness of complex biological systems. The human brain epitomizes a biological context in which function and dysfunction is defined by patterns of information flow, which is reflected by the intercellular exchange of defined molecular signals.

Cellular interactions are mostly thought of as being organized into molecular pathways of autocrine, juxtacrine, paracrine, or endocrine nature (depending on the intercellular distances). According to this paradigm a target cell is subjected to iterations of individual receptor-ligand recognition events, and their networks, many of which are now well-characterized (e.g., in the case of hormones, neurotransmitters, growth factors, and membrane molecules and their respective receptors) (Avraham and Yarden, 2011).

This compelling model, however, has long eclipsed some other “non-conventional” forms of cellular communication (Mittelbrunn and Sanchez-Madrid, 2012). Indeed, it is increasingly understood that cells also produce combinatorial messages contained in cellular and membrane fragments including entities usually regarded as confined to insoluble, intracellular compartments (cytoplasm, nucleus, transport vesicles). Such bursts of multimolecular information may be received by other cells and lead to a change in their functional state along with elements of their molecular identity.

Indeed, this complex form of intercellular communication may have an ancient ancestry. This is exemplified by the phenomenon of horizontal gene transfer (HGT), which is implicated in certain forms of speciation and organismal symbiosis (Choi and Kim, 2007; Court et al., 2008). Intriguing remnants of such relationships include the insertion (and expression) of the entire genome (DNA) of the intracellular prokaryote *Wolbachia* in its carrier insect cell. In this sense cell fusion, phagocytosis, and formation of viral particles by higher organisms could be regarded as relics of intercellular integration developed during early evolution, a process “rediscovered” in the course of various physiological and pathological processes in higher species (Sinkovics, 2011).

The horizontal transfer of molecules is also known to occur between human cells, including those in the brain. This process may be executed through several different mechanisms involving rearrangements within specialized plasma membrane domains, and formation of direct cell–cell contact sites. Examples of such processes include formation of intercellular junctions, membrane swapping (trogocytosis), cellular synapses, extension of tunneling nanotubes or cytonemes, and other mechanisms acting mostly between adjacent cells (Belting and Wittrup, 2008). However, cells also posses the capacity to exchange membrane fragments and associated complex molecular signals over longer distances (often systemically) subsequent to the formation and release of organelle-like structures often referred to as extracellular vesicles (EVs), which are the main focus of this article.

## Biogenesis and properties of extracellular vesicles

EVs shed from individual cells are molecularly complex and often highly heterogeneous. Although there is no consensus as to the exact mechanisms that govern EV formation and their nomenclature, the most common descriptions point to at least four distinct vesiculation pathways. Thus, apoptotic cellular breakdown leads to the release of large EVs (>1000 nm in diameter) known as apoptotic bodies (AB) that contain cytoplasmic and membrane material, genomic DNA, and organelles. Even larger particles (large oncosomes, 1000–10,000 nm) are generated from plasma membrane blebs, as a by-product of the amoeboid motility exhibited by certain types of cancer cells (Di Vizio et al., 2009). Through a similar membrane blebbing mechanism various phagocytes, microglia, platelets, and cancer cells emit smaller EVs referred to as microvesicles (MVs), microparticles, shed vesicles, or ectosomes (usually 100–1000 nm in diameter) (Thery et al., 2009). In this case, the stimulation with biological agonists triggers calcium fluxes, regional loss of phospholipid asymmetry in the plasma membrane, exposure of phosphatidylserine (PS), followed by changes in membrane-cytoskeleton contacts, formation of membrane curvature, and vesicle scission (Piccin et al., 2007). In microgial cells, this process involves acidic sphingomyelinase (Asmase) and activation of intracellular kinase cascades (Bianco et al., 2009). A similar mechanism is also responsible for the extracellular release of certain integral membrane receptors such as tissue factor (TF), the main trigger of blood coagulation expressed by phagocytes and cancer cells, including glioma (Yu and Rak, 2004; Del Conde et al., 2005). Depending on their source, MVs may also contain cellular lineage markers, high levels of surface PS, integrins, cannabinoid receptor (CB1), matrix metalloproteinases (MMPs), TF, and other membrane-related entities defining their unique biological features along with lipids and possibly nucleic acids (Dolo et al., 2005; Bianco et al., 2009; Camussi et al., 2010; Lee et al., 2011a).

A relatively well-studied and distinct form of vesiculation involves the formation of exosomes. These EVs are believed to be generated intracellularly, as the so-called intraluminal vesicles (ILVs). These secondary vesicular structures emerge within larger endosomal vesicles described as multivesicular bodies (MVBs) (Simons and Raposo, 2009; Thery et al., 2009; Mathivanan et al., 2010). Formation of MVBs represents a step in membrane receptor signaling and processing cascade, which involves receptor internalization controlled by the endosomal sorting complex required for transport (ESCRT). This multimolecular apparatus controls the intracellular trafficking of membrane receptors between cell surfaces, endosomes and pathways of lysosomal destruction, or recycling (Williams and Urbe, 2007). It is believed that in some instances MVBs take an alternative path and are instead redirected to the plasma membrane in such a way as to allow the extracellular release of ILVs (as exosomes) (Trajkovic et al., 2008). Exosomes are relatively small (30–100 nm), rich in tetraspanins (CD63, CD9), Rab proteins, and other cargo including nucleic acids (Valadi et al., 2007; Thery et al., 2009).

## Extracellular vesicles as vehicles of intercellular communication and molecular exchange

Emission of EVs constitutes a natural multiplexing mechanism whereby several molecules may be assembled, protected, and released from cells regardless of their compatibility with the classical secretory pathways. Indeed, vesiculation represents the key mechanism whereby proteins lacking a signal peptide (e.g., interleukin 1 beta–IL1β), or located in non-secretory cellular compartments (e.g., nuclear proteins) may reach the extracellular space (Bianco et al., 2009). Consequently, various proteins and nucleic acids are incorporated into EVs, often in concentrations higher than those found in parental cells. While the astonishing scope of this “packaging and shipment” process has been reviewed in the recent literature and cataloged in specialized databases (Exocarta) (Ratajczak et al., 2006b; Valadi et al., 2007; Thery et al., 2009; Mathivanan et al., 2012), the related mechanisms remain elusive, with only limited but intriguing insights (Bolukbasi et al., 2012).

The functional implications of cellular vesiculation can, at least to some extent, be inferred from the repertoire of EV-associated bioactive molecules. While EVs may contain high concentrations of soluble mediators (interleukins, growth factors, chemokines), their unique role in cell–cell interaction is thought to stem largely from their content of transmembrane, cytoplasmic and nuclear proteins, lipids, mRNA, miRs, genomic DNA sequences (Ratajczak et al., 2006b; Valadi et al., 2007; Mause and Weber, 2010; van der Vos et al., 2011).

**Figure 1:**
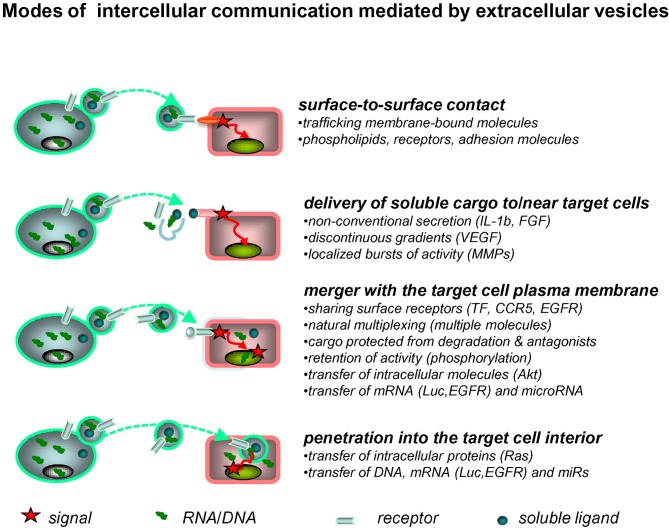
**Extracellular vesicles as mediators of intercellular communication.** Exchange of molecular information between cells may be mediated by EVs in several ways. Thus, surface receptors of EVs may interact directly with counter-receptors on the surface of a target cell. The latter may also come into contact with the bioactive inner cargo of EVs upon their pericellular rupture. EVs may also merge with the plasma membrane of the target cell, or penetrate into its interior via endocytosis, or other processes, to release their content of proteins and nucleic acids into the intracellular compartments (see text for details).

EVs interact with various target cells through several mechanisms (Figure 1). The fate of EVs involved in such interactions may entail either a simple surface contact with the target cell, e.g., via receptor-ligand bridges, or several other processes. Those include rupture of the EV membrane leading to pericellular release of their cargo, and a burst of paracrine activity (Taraboletti et al., 2006). However, EVs may also reach the interior of their target cells by fusion with their plasma membranes, or through an endocytosis-like engulfment of the entire vesicle. In these instances, the bioactive cargo of EVs becomes released inside the target cell, and thereby may interact with their regulatory apparatus including adapter proteins and signaling circuitry (Al-Nedawi et al., 2009b). The efficiency and consequences of these cell-EV-cell interactions may depend on the nature of the cells involved and on the surrounding microenvironment (hypoxia, inflammation, acidity), all of which may control the emission, cargo, and uptake of EVs. In this regard, the brain represents a unique site for EV-mediated interactions.

## Extracellular vesicles in the brain microenvironment

There are several cellular sources of EVs that may enter the interstitium, fluid spaces, and other compartments of the brain microenvironment. For example, EVs are normally present within the vascular system and may readily enter the brain microcirculation. In the absence of disease, those are mainly EVs (microparticles) released from activated blood platelets (Key et al., 2010) or inflammatory cells. In addition, other extracranial sources may contribute to the EV pool in the brain vasculature, including EVs generated by peripheral inflammatory cells, endothelium, or distant cancer cells (Smalheiser, 2009; Lee et al., 2011a). While certain formulations of dendritic cell exosomes have been shown to penetrate the blood-brain barrier (BBB) (Alvarez-Erviti et al., 2011), there is no conclusive evidence for a free and consequential exchange of naturally occurring EVs between brain parenchyma and peripheral tissues. This could likely take place, however, at sites of injury, or in hyperpermeable vessels associated with tumor growth. Circulating EVs may also freely interact with brain endothelial cells, and thereby potentially affect their state and function, or participate in thrombosis and other forms of vascular pathology (Chen et al., 2011). While many of these possibilities are poorly studied, EVs and their associated ectonucleotidases have been implicated in cytoprotective and repair events once BBB has been disrupted (Ceruti et al., 2011).

EVs have also been implicated in various processes involving brain parenchymal cells. For instance, neuronal stem cells (NSCs) produce EVs containing the CD133 progenitor marker (Marzesco et al., 2005). Exocytosis is also well-described in differentiated neurons and may impact their communication with non-neuronal cells (Smalheiser, 2009). Indeed, neurons contain MVBs, the structural precursor of exosomes (von Bartheld and Altick, 2011), and these EVs are also found in supernatants of corresponding cell cultures (Faure et al., 2006). Similarly, normal glial cells, such as astrocytes release EVs into their surroundings. In this manner glial-derived glutamate may reach and act on its receptors associated with adjacent neurons (Bergersen and Gundersen, 2009). Astrocytes also shed EVs containing mitochondrial DNA, but the significance of this process is presently unclear (Guescini et al., 2010). Oligodendrocytes were found to produce exosomes (Kramer-Albers et al., 2007), a process that relies on a specific pathway involving neutral sphingomyelinase (Nsmase) (Trajkovic et al., 2008). These EVs are then selectively taken up by brain microglial cells, which are postulated to provide a constitutive mechanism for exosome clearance within the milieu of the brain (Fitzner et al., 2011). Microglial cells themselves emit EVs containing cytokines (Potolicchio et al., 2005), a process recently implicated in neuroinflammation (Bianco et al., 2009). In this regard, Verderio and colleagues described a regulatory pathway involving Asmase, which controls ATP stimulated release of EVs from microglial cells. In this fashion EV-associated IL-1β, which lacks secretory signal peptide, can be liberated from microglia and act as stimulator of phagocytosis, which is required for clearance of ATP emitting damaged cells (Bianco et al., 2009). Microglial EVs also play a previously unsuspected role in neuronal synaptic activity (Antonucci et al., 2012). Indeed, due to this emerging network of EV-mediated interactions in the brain the emission and content of various vesicles was recently proposed to serve as a putative biomarker for neurological disorders (Colombo et al., 2012). It remains to be established to what extent EV production, trafficking, and uptake contribute to the pathogenesis of these conditions, and whether their release also has notable systemic consequences.

## Biological effects of extracellular vesicles in cancer

The process of cellular vesiculation is hijacked and distorted during malignant transformation and contributes to the phenotype of cancer cells and their associated stroma. This has been documented in several different disease settings, and reviewed extensively in recent literature (Ratajczak et al., 2006b; Thery et al., 2009; Camussi et al., 2010; Rak and Guha, 2012). The role of EVs in cancer is often a subject of generalizations, which will likely evolve to more disease-specific considerations as the underlying processes become better understood. It is reasonable to predict that EVs may differ in their type and relative role in the pathogenesis of different cancer types and disease subtypes, also as a function of such variables as host genetic background, in a similar manner as this applies to other effector mechanisms associated with malignancy (e.g., angiogenesis or metastasis) (Rohan et al., 2000; Hunter, 2006; Phillips et al., 2006). Moreover, the contribution of EV release is difficult to formally demonstrate due to the scarcity of suitable loss-of-function models *in vivo*, where tumor progression could be rigorously examined in the presence and absence of vesiculation. Nonetheless, correlative studies provide compelling evidence for the involvement of EV generation and exchange in several aspects of neoplasia.

Amongst the more extensively studied aspects of vesiculation is the involvement of EVs in cancer coagulopathy. Indeed, one of the first description of EVs was related to procoagulant microparticles emanating from activated platelets (“platelet dust”) (Wolf, 1967). This “shedding” mechanism has since been implicated in prothrombotic, proangiogenic, and prometastatic events in cancer (Baj-Krzyworzeka et al., 2002; Janowska-Wieczorek et al., 2006). Seminal studies of Dvorak and colleagues revealed extensive shedding of procoagulant TF-containing microvesicles from cancer cells (Dvorak et al., 1983). Numerous subsequent analyses interrogated the relevance of this process in cancer biology (Yu et al., 2005), progression (Tesselaar et al., 2007), and paraneoplastic (prothrombotic) syndromes (Burnier et al., 2009; Aharon and Brenner, 2010; Khorana, 2010; Zwicker, 2010).

Production of exosomes by cancer cells has been frequently implicated in anticancer immunity (Wolfers et al., 2001). In this regard, both positive and negative effects of circulating exosomes were proposed to regulate antitumor responses (Wolfers et al., 2001; Taylor and Gercel-Taylor, 2005; Liu et al., 2006; Valenti et al., 2007). Among the most interesting examples is the discovery of exosomes containing Fas ligand, which could effectively destroy Fas receptor—expressing cytotoxic effector T cells before they could reach cancer cells (Abusamra et al., 2005). There are also indications that exosomes derived from glioblastoma (GBM) cells may exert immunomodulatory effects on monocytes (de Vrij et al., 2012).

EVs may harbor molecular mediators of drug resistance and transfer them between cells. This may lead to the exchange of pro-survival proteins (Al-Nedawi et al., 2008), molecular drug efflux pumps (e.g., P-glycoprotein/MDR1) (Jaiswal et al., 2012) or other cargo. A similar exchange of plasma membrane fragments containing drug resistance molecules may also occur upon cell–cell contact, through a mechanism known as trogocytosis (Rafii et al., 2008).

Cancer-derived EVs have also been implicated in metastasis. For example, recent experimental data suggests that exosomes cooperate with other pathways in the formation of pre-metastatic niches and promote hematogenous metastases at distant sites (Jung et al., 2009; Grange et al., 2011; Peinado et al., 2011). Similarly, the influence of exosomes has been observed in the context of lymphatic dissemination (Hood et al., 2011) and local invasion (Hendrix et al., 2010).

EVs may also influence disease dissemination through their impact on the vascular system including angiogenesis. In this regard, both host and tumor-derived EVs appear to possess an array of proangiogenic activities attributed to several elements of their cargo. Thus, EVs emanating from platelets (Janowska-Wieczorek et al., 2005) and endothelial progenitor cells (Deregibus et al., 2007) have the ability to stimulate the angiogenic program in resident endothelial cells. In another study, tetraspanin (Tspan8)-containing exosomes emanating from certain experimental cancer cells were found to elicit a systemic proangiogenic state in mice harboring the corresponding tumors (Gesierich et al., 2006). It is noteworthy that EVs may contain high concentrations of soluble angiogenic molecules such as IL-8, VEGF, FGF (Taraboletti et al., 2006; Skog et al., 2008) as well as proangiogenic matrix metalloproteinases (MMP9) and their regulators (CD147). In this manner, EVs may deliver bursts of activity to sites of blood vessel formation, in and around the tumor, or at distant sites (Taraboletti et al., 2006). EVs may also carry normally insoluble angiogenesis regulators such as delta like 4 (Dll4), the cellular ligand of Notch. Presentation of Dll4 to Notch in the EV-associated form alters the biological activity of this angiogenic pathway (Sheldon et al., 2010). Moreover, interaction of EVs with target cells may modulate their angiogenic phenotype, either through EV-cell contact, or by horizontal transfer of signaling molecules (Ratajczak et al., 2006b; Al-Nedawi et al., 2008; Skog et al., 2008; Al-Nedawi et al., 2009a). While the requirement for such EV-mediated communication for the onset and regulation of angiogenesis is not fully explored, a multitude of angiogenic effectors are already known to be released via cellular vesiculation pathways, which likely influences their activity (e.g., by changing their spatial distribution and gradients) (Mause and Weber, 2010).

Several additional effects of EVs in cancer are also of considerable interest. This includes communication and reprogramming events that may occur through contact between cancer cells and EVs emanating from stem cells, as originally observed by Ratajczak and colleagues (Ratajczak et al., 2006a). Other types of progenitor-like cells are also known to shed EVs (Milsom et al., 2008; Collino et al., 2010), and this may include tumor initiating (cancer stem) cells (TICs) identified in several malignancies including brain tumors (Stiles and Rowitch, 2008). It is conceivable that TICs may possess the capacity to reprogram activities of other cells via the exchange of EVs.

## Neovesiculation and oncosomes

Vesiculation of cancer cells may take several aberrant forms including quantitative increases in EV emission, changes in their size, structure, and molecular composition, as well as altered biological activity. Some of these anomalies may be a function of disease-related aberration in the EV biogenesis pathways, changes we collectively refer to as *neovesiculation*.

Several mechanisms have been described that effectively differentiate the EV emission by cancer cells from that of their corresponding non-transformed counterparts. For instance, in prostate cancer cells, deregulation of the Akt pathway, growth factor stimulation (EGF), and loss of the diaphanous related formin 3 (DRF3) leads to the acquisition of a cellular phenotype associated with invasiveness, amoeboid motility, and unique form of neovesiculation. The latter is characterized by formation of very large membrane blebs on the cell surface, and their subsequent scission as the aforementioned unusually large EVs (*large oncosomes*). Large oncosomes exhibit biological activities consistent with their content of signaling molecules, and their formation may be viewed as a hallmark of increased prostate cancer aggressiveness linked to a loss of a putative tumor suppressor (DRF3) (Di Vizio et al., 2009).

The term *oncosomes* was originally coined to reflect another distinct feature of tumor cell-derived EVs, namely their ability to carry cancer-specific mutant proteins and nucleic acids, the very drivers of oncogenic transformation and hitherto regarded as confined to cancer cells. Although oncogenic mutations are normally thought of as propagating along vertical clonal hierarchies, the release of their containing molecules (oncoproteins and nucleic acids) as cargo of EVs suggests that mutant gene products may traffic horizontally between cells. In this manner, transforming signals could be shared amongst wider cellular populations including indolent, normal, and unrelated (heterotypic) cells (Al-Nedawi et al., 2008). Notably, oncogene-containing EVs were found in the interstitium, body fluids, and circulating blood in tumor bearing animals and cancer patients (Al-Nedawi et al., 2008; Skog et al., 2008). Through this mechanism distant organ sites may become exposed to transforming activities, including cells within putative metastatic niches, stem cell reservoirs, and regulatory cell populations within the vascular system and bone marrow (Rak and Guha, 2012). Although several long and short range biological effects of EVs have already been described in various cancer settings (Al-Nedawi et al., 2008; Ghosh et al., 2010; Antonyak et al., 2011), the specific role of oncogenic molecules in these events is still to be formally demonstrated *in vivo*. Several types of EVs may contribute to the extracellular release of oncogenic cargo from cancer cells, including large and small oncosomes and exosome-like vesicles (Al-Nedawi et al., 2008; Skog et al., 2008; Al-Nedawi et al., 2009a; Graner et al., 2009). In this regard cancer cell apoptosis represents a distinct mechanism, whereby cellular remnants (AB) may serve as unique vehicles for vesicular trafficking of mutant DNA sequences in the pericellular milieu (Holmgren et al., 1999).

## The transforming cargo of oncosomes

Oncosomes may harbor several types of cancer-related molecules including active oncoproteins, oncogenic transcripts, transforming miR species, and genomic sequences containing mutant oncogenes. Likewise, wild type or mutant tumor suppressors (proteins and nucleic acids), and molecules affecting genetic stability (e.g., retrotransposons) have also been identified in the cargo of cancer-derived EVs, as reviewed in the recent literature (Ratajczak et al., 2006b; Muralidharan-Chari et al., 2010; van der Vos et al., 2011; Rak and Guha, 2012).

Amongst the best described examples of oncoproteins found in the cargo of cancer-derived oncosomes are members of the ErbB/HER family of receptor tyrosine kinases (RTKs), such as activated (phosphorylated) EGFR and its constitutively active mutant EGFR variant III (EGFRvIII) (Al-Nedawi et al., 2008, 2009a, 2010). Breast cancer cells have been found to shed EVs containing HER-2 protein, another member of the EGFR family (Koga et al., 2005). Different cancer cell lines shed EVs containing other oncoproteins including myr-AKT (Di Vizio et al., 2009), LMP1 (Meckes et al., 2010), Ras (Lee and Rak, 2011, unpublished observation), including mutant K-ras (Franklin et al., 2012), BRAF/V600E (Ramachandran et al., 2011), PDGFR, beta-catenin, c-Met, and several others (Al-Nedawi et al., 2010). EVs may also contain tumor suppressor proteins (e.g., PTEN) (Al-Nedawi et al., 2010) and their potential role in horizontal modulation of the malignant phenotype is a subject of an ongoing interest.

Oncogenic nucleic acids have also been identified in the cargo of various EVs, including transcripts for the various aforementioned oncoproteins (Skog et al., 2008; Graner et al., 2009). As mentioned earlier, AB may carry DNA sequences associated with the Epstein-Bar virus-related oncogenes (EBNA1, EBER), as well as those encoding oncogenic H-ras and Myc (Holmgren et al., 1999; Bergsmedh et al., 2001). Cell culture medium and serum of mice harboring human medulloblastoma (MB) xenotransplants may contain EVs with encapsulated DNA corresponding to the amplified oncogenic c-Myc sequences (Balaj et al., 2011), while plasma of colorectal cancer patients was found to contain functional circulating DNA encoding mutant K-ras (Garcia-Olmo et al., 2010).

Pioneering work of several investigators provided ample evidence as to the presence of multiple miR species in the cargo of EVs emanating from various cell types (Ratajczak et al., 2006b; Valadi et al., 2007; Skog et al., 2008; Taylor and Gercel-Taylor, 2008). Much research on miR detection in samples of blood or cerebrospinal fluid (CSF) collected from cancer patients has focused on the simultaneous isolation of all circulating miRs including their protein- and microparticle-associated fractions (Chen et al., 2012). Taylor profiled miRs in both the tumor tissue and serum-derived, tumor-specific exosomes collected from ovarian cancer patients. Those miRs (miR-21, miR-141, miR-200a, miR-200c, miR-200b, miR-203, miR-205, and miR-214) that were present in both the tumor and exosomes, and which had been previously identified as overexpressed in human ovarian cancer were then validated by qRT-PCR demonstrating a direct correlation between the miR signature of the tumor and that of the tumor-derived exosomes (Taylor and Gercel-Taylor, 2008). All of these miRs were significantly elevated in exosomes collected from patients diagnosed with early and late stage ovarian cancer compared to benign ovarian disease; however, miR-200c and miR-214 were also specifically present in higher copy numbers in late stage malignancies. The levels of circulating let-7a and miR-195 are significantly elevated in plasma samples collected from breast cancer patients compared to healthy women (Heneghan et al., 2010). These miRs were also overexpressed in the tumor relative to normal tissue. Interestingly, both aforementioned miRs may also act as biomarkers of therapeutic response, as post-operative levels were comparable to blood samples collected from healthy women. In prostate cancer patients circulating miR-141 is higher compared to healthy individuals (Mitchell et al., 2008). Furthermore, circulating miR-141 and miR-375 (also elevated in prostate cancer specimens compared to normal tissue) are associated with metastatic disease (Brase et al., 2011). Profiling studies of miRs found in large oncosomes in prostate cancer revealed a pro-invasive signature. A more comprehensive list of circulating miRNAs that may act as diagnostic and/or prognostic biomarkers can be found in past reviews (Kosaka et al., 2010; Cortez et al., 2011).

At least some of these miR species, may possess oncogenic and tumor suppressive characteristics (Garzon et al., 2010). Indeed, we observed that MB cells engineered to express miR-520g shed EVs containing this miR into culture media and the blood of xenograft bearing mice (D'Asti et al., 2012). Mir-520g acts as an oncogene in these and other neuroectodermal tumors (Li et al., 2009). Several other putative oncomirs have also been detected in the cargo of EVs released from human GBM cells including let7a, miR-16-1, miR-92, and miR-21 (Skog et al., 2008).

## Biological consequences of oncosome production

**Figure 2:**
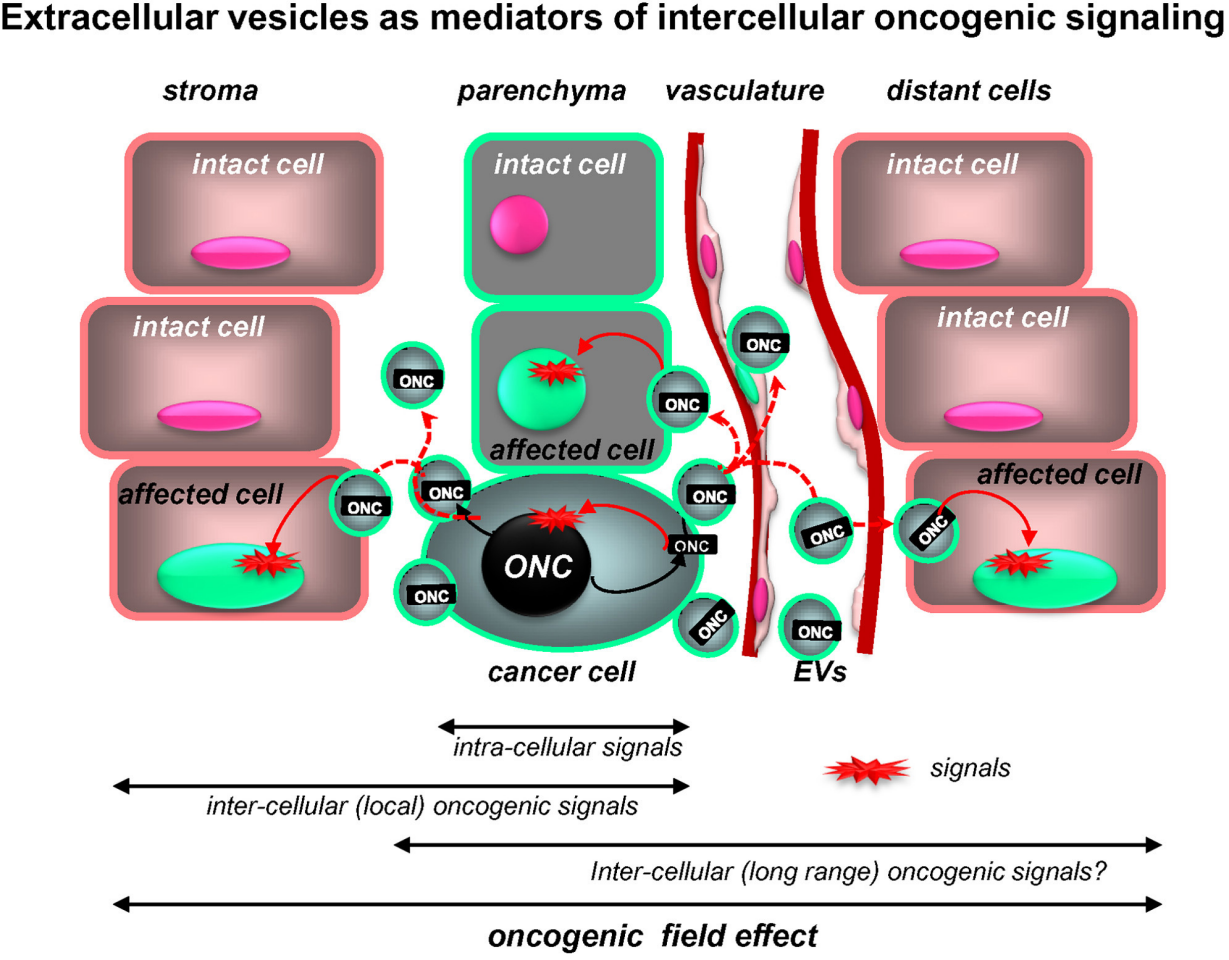
**Extracellular vesicles as putative mediators of the intercellular propagation of oncogenic signaling.** While intracellular signaling pathways elicited by mutant oncogenes (*ONC*) are increasingly well understood, oncoproteins (ONC) may also operate outside of the confines of cancer cells due to intercellular trafficking of their containing EVs (oncosomes). Uptake of this material by proximal non-transformed cells and at distant organ sites may trigger downstream oncogenic signals in these recipient cells and alter their phenotype and behavior. Thus, intercellular trafficking of oncoproteins (and nucleic acids) extends the range of oncogenic signaling beyond the boundaries of cells harboring the original mutation (see text for details).

The biological significance of the EV-mediated release of oncogenic molecules is usually inferred from their inherent transforming activity coupled with the ability to undergo intercellular trafficking. While this is an intriguing possibility, there is no formal and conclusive *in vivo* evidence in support of the absolute requirement or the rate-limiting involvement of vesiculation in key aspects of cancer progression. Nonetheless, proof-of-principle experiments *in vitro* or in mouse models suggest several potential pathogenetic mechanisms and the existence of the unexpected, intercellular dimension of oncogenic signalling (Figure 2).

Thus, EVs containing oncogenic EGFRvIII are capable of transferring this oncoprotein into indolent glioma cells, in which this influence activates the canonical MAPK and AKT signaling pathways. The biological consequences of this “ectopic signaling” include augmentation of soft agar colony formation, production of angiogenic factors, and changes in gene expression (Al-Nedawi et al., 2008). Tumor cell-derived EVs can also mediate the transfer of EGFR to endothelial cells inducing aberrant signaling and autocrine activation of VEGF receptors (VEGFR2). Similar quasi-transforming *in vitro* consequences are associated with the cellular uptake of EVs containing activated AKT and LMP1 proteins (Di Vizio et al., 2009; Garnier et al., 2010). Exosomes can also mediate a transfer of oncogenic K-ras between aggressive and indolent colorectal cancer cell lines, causing transformation-like changes (Franklin et al., 2012).

Even more dramatic outcomes were observed when non-tumorigenic NIH3T3 fibroblasts were exposed to EVs derived from invasive breast cancer cells containing tissue transglutaminase (tTG) and fibronectin (FN). Uptake of this material *in vitro* and *in vivo* led to overt transformation and tumorigenic conversion of the NIH3T3 recipients (Antonyak et al., 2011). Similarly, DNA sequences containing oncogenic K-ras gene were detected in association with particles circulating in blood of colorectal cancer patients. Again, the uptake of this material by NIH3T3 cells resulted in the onset of their tumorigenic phenotype. This phenomenon was postulated to play a role in the remote transformation of normal cells and formation of distant outgrowths, a process termed “genometastasis” (Garcia-Olmo et al., 2010). Many of these observations are consistent with the pioneering work of Holmgren and colleagues who originally demonstrated that the uptake of oncogenic DNA sequences (H-ras, c-Myc) contained in tumor cell-derived AB (EVs) may lead to the expression of the respective oncoproteins and tumorigenic phenotype in non-transformed recipient cells (mostly NIH3T3 fibroblasts) (Holmgren et al., 1999; Bergsmedh et al., 2001).

While the aforementioned observations raise the spectre of EV-mediated widespread dissemination of oncogenic material and horizontal transformation of normal cells, the likelihood and scope of such events requires some qualification. First, the half-life of oncoproteins and their transcripts in recipient cells is probably somewhat limited due to breakdown and dilution during cell division. Second, cells differ in their ability to take up, retain, and utilize the EV-related material. In fact, shedding of EGFR and other oncoproteins as EV cargo may represent a mechanism of removal of these overabundant molecules from their parental cancer cells, and could be reiterated in EV recipients. Aggregation of proteins at the plasma membrane may serve as a trigger for such protective shedding processes (Shen et al., 2011; van der Vos et al., 2011). Third, the biological effects of the uptake of active oncoproteins by non-transformed cells may not always be tantamount to cellular transformation. This is because normal cells (unlike immortalized NIH3T3 fibroblasts) retain a repertoire of tumor suppressors that may activate apoptotic or senescence programs in the presence of protracted oncogenic signaling, a phenomenon known as “oncogenic stress response” (Serrano et al., 1995). Even established but indolent cancer-derived cells do not necessarily undergo overt tumorigenic conversion upon the uptake of EGFRvIII-containing oncosomes (our unpublished observation), and additional genetic events or molecular predispositions may be required for such a change to take place. However, the potential that at least some cells (e.g., stem cells, premalignant cells, or dormant cancer cells) may be susceptible to malignant conversion via oncosome-mediated molecular transfer cannot be excluded at this time. It is also likely that more transient phenotypic changes (increased angiogenic potential, cellular activation, stress responses) may result from exposure of various normal and indolent cells to circulating oncosomes in cancer patients.

## Modulation of cancer cell vesiculation by microenvironment, stress, and differentiation pathways

Oncogenes and tumor suppressors do not only function as cargo, but also as a part of the regulatory circuitry that controls cellular vesiculation in cancer. Some recent examples to this effect include the modulation of exosome production by p53 tumor suppressor (Yu et al., 2006) and its target known as TSAP6 (Lespagnol et al., 2008). On the other hand, loss of p53 expression may enhance EV-mediated emission of TF from colorectal cancer cells. Oncogenic Ras, EGFRvIII, constitutively activated AKT (myr-AKT) exhibit vesiculation-inducing effects in various settings (Yu et al., 2005; Al-Nedawi et al., 2008). These intrinsic effects epitomize the link between various intracellular pathways and regulation of EV production in response to external stresses and stimuli. For instance, the aforementioned p53-regulated EV emission occurs prominently in cells undergoing radiation responses, i.e., when this suppressor protein is induced and plays an important biological role (Yu et al., 2006). Analogous alterations in EV production could be expected in cells exposed to other forms of genotoxic or microenvironmental stress, hypoxia, metabolic deprivation, or contact with inflammatory mediators (Svensson et al., 2011), with possible involvement of pathways containing proto-oncogenes and tumor suppressors. Similarly, exposure of various cells to high concentrations of exogenous EGFR ligands (EGF, TGFα) often triggers robust cellular vesiculation (Di Vizio et al., 2009; Garnier et al., 2012).

Cancer cells form clonal hierarchies in which oncogenic, differentiation, and extracellular stimulation pathways blend to control cellular composition and behavior. This includes pathways that define cellular stemness and trans-differentiation events, of which epithelial-to-mesenchymal transition (EMT) represents an important example. EMT is a process whereby cancer cells of epithelial or ectodermal origin (including neuroectodermal cells) transiently acquire a mesenchymal phenotype (e.g., vimentin positivity), as well as more motile and tumor initiating properties (Mani et al., 2008), all of which are implicated in aggressive and metastatic growth (Thiery et al., 2009). Several molecular events are capable of inducing EMT, including cooperation between Ras and TGFb signalling pathways, activation of the MET receptor, induction of several EMT-related transcription factors (e.g., YB1, Twist, or Brachyury) (Fernando et al., 2010), blockade of E-cadherin, and other changes (Thiery et al., 2009).

In A431 squamous cell carcinoma cells harboring an amplified *EGFR* gene, stimulation with EGFR ligands (TGFα) coupled with blockade of E-cadherin results in an EMT-like state characterized by the onset of vimentin expression, and spindle morphology, as well as internalization of cell surface receptors, and a profoundly altered vesiculation profile. The latter includes the overall increase in EV emission, increase in EV-associated TF antigen, as well as elevated emission of exosome-like particles (Garnier et al., 2012). These changes are associated with greater tumor initiating capacity, as measured by increased numbers of metastatic colonies resulting from intravenous injection of A431 cells *in vivo* (Milsom et al., 2008). A reflection of some of these changes could also be found in the proteome of EVs emitted by cells that have entered the mesenchymal state as a result of expression of oncogenic EGFR (Garnier et al., 2012) or H-ras (Tauro et al., 2012).

Interestingly, molecular elements of the EMT-inducing machinery may not only modulate cellular vesiculation, but also are often found in the EV cargo. This has been observed in the case of YB1 (Frye et al., 2009), EGFR, and MET (Al-Nedawi et al., 2008, 2010). Since EMT often co-segregates with the elevated tumor initiating (stem cell) capacity of cancer cells, it is possible that the accompanying changes in vesiculation may contribute to this process in some way; for example, by conditioning the niche environment, influencing the adjacent host cells (Ratajczak et al., 2006a), modulating sites of metastasis (Hood et al., 2011), or impacting the vasculature (Gesierich et al., 2006). Indeed, a link between cancer stem cell vesiculation and angiogenesis has recently been described (Grange et al., 2011). It is presently unclear whether these processes involve intercellular transfer of oncogenic molecules.

## Oncosomes in brain tumors

As in the case of other malignancies, oncogenic proteins and nucleic acids may be emitted from brain cancer cells as EV cargo (Al-Nedawi et al., 2008; Skog et al., 2008; Balaj et al., 2011). Likewise, oncogenic signaling intermediates and effector molecules may be present in EVs produced by different types of primary and secondary brain tumors, their surrounding parenchyma, microglia, stroma, vasculature, and blood cells. The scope of these processes and their biological impact, however, are far from understood and only a limited number of examples have been published to date (Al-Nedawi et al., 2008; Skog et al., 2008; Graner et al., 2009; Balaj et al., 2011).

Initial reports suggested that biologically active, phosphorylated, and oncogenic EGFRvIII protein is contained in the cargo of small (100–400 nm) EVs produced by EGFRvIII-transformed GBM cells, and this material is emitted into the culture media and plasma of tumor xenograft-bearing mice (Al-Nedawi et al., 2008). These studies documented the aforementioned EV-mediated transfer of EGFRvIII activity to indolent U373 glioma cells and the resulting upregulation of VEGF, BclXL, and changes in levels of other EGFR target genes, as well as increased soft agar colony forming capacity. Co-injections of growth arrested (Mitomycin C-treated) EGFRvIII expressing EV donor cells with GFP-tagged indolent EV recipient glioma cells revealed the expected intercellular transfer of the EGFRvIII immunofluorescence *in vivo*. However, no overt tumorigenic conversion of the indolent cells has been recorded in these experiments [(Al-Nedawi et al., 2008, 2010) and our unpublished data]. The expression of EGFRvIII, EGFR, PDGFR, MET, PTEN, and other GBM-related oncogenic and tumor suppressive proteins was also noted in other models of high grade glioma (U87, U87vIII, U87-PTEN) (Al-Nedawi et al., 2010; Lee et al., 2011b). In addition, a recent study of the phosphoproteome associated with EVs shed by the U373vIII GBM cell line harboring mutant EGFRvIII revealed a rich repertoire of proteins that have undergone this activating post-translational modification, including molecules with oncogenic, signaling, and gene-regulatory potential. This list includes phosphorylated membrane receptors (EGFR, HER2, MET), intracellular protein kinases (PKC, MEK1, Raf1), regulators of apoptosis (BAD), transcription factors (Jun, CREB1), regulators of protein translation (eIF4E, eIF2A), histones (H2B, H3.3), and DNA binding proteins (steroid receptors) (Al-Nedawi et al., 2010). In agreement with these findings, the recent proteomic analysis of GBM-derived exosomes documented the presence of EGFRvIII in samples isolated from culture supernatants and patient plasma (Graner et al., 2009).

Recent elegant studies by Skog, Breakefield and their colleagues brought to light the presence of oncogenic nucleic acids in EVs derived from brain tumors (Skog et al., 2008; Balaj et al., 2011; van der Vos et al., 2011). For example, the EGFRvIII transcript was present in the cargo of EVs isolated from culture medium of primary GBM cell isolates, and in plasma of GBM patients (Skog et al., 2008) as well as in corresponding circulating platelets that appear to take up GBM oncosomes (Nilsson et al., 2011). Notably, levels of the EGFRvIII mRNA signal in plasma were reduced upon surgical tumor de-bulking, which confirmed the tumor-related origin of this material. These investigators have also demonstrated the functionality of the EV-associated mRNA in driving gene expression (luciferase) upon intercellular transfer. These experiments documented robust biological effects of GBM-derived EVs especially as stimulators of cellular growth and endothelial morphogenesis (Skog et al., 2008). Molecular profiling of GBM-associated EVs unveiled a rather astonishing wealth of molecular species, including mRNA, non-coding RNA (multiple miRs), and proteins, some of which were enriched in EVs in comparison to parental cells (Skog et al., 2008). The mechanism of cargo assembly and molecular enrichment during EV biogenesis remains unclear, but in the case of mRNA this process may depend on a specific “zipcode-like” 25 nucleotide sequence at the 3'UTR. This motif is thought to selectively guide certain transcripts to the regions of EV biogenesis with the help of miR-1289 (Bolukbasi et al., 2012). Various mRNA sequences were detected in EVs isolated from plasma of an independent cohort of GBM patients (Noerholm et al., 2012). With a few aforementioned exceptions, studies do not provide a conclusive picture as to the biological activity *in vivo* and the oncogenic potential of EV-associated molecules found in plasma of patients with GBM, but this remains a disturbing possibility. Moreover, brain tumor cells produce EVs containing oncomirs. This includes the emission of miR-520g, which is a part of the 19q13.41 amplicon associated with a subset of supratentorial primitive neuroectodermal tumors (sPNET) (Li et al., 2009). As mentioned earlier, cells transfected with the corresponding pre-miRNA gene release EVs containing miR-520g (D'Asti et al., 2012). The emission and biological role of other oncomirs involved in primary and secondary brain tumors have not been studied.

EVs isolated from viable brain tumor cells have also been recently shown to contain functional DNA sequences (exoDNA). GBM cells emit EVs that contain retrotransposons and are capable of mediating their transfer to recipient endothelial cells. Several MB cell lines expressing the amplified c-Myc oncogene emit the corresponding genomic sequences as EV cargo, both *in vitro* and *in vivo* (Balaj et al., 2011). It is not clear whether these EVs possess Myc-related biological activity. The corresponding mechanisms by which the relatively large genomic amplicon sequences may be processed into single stranded DNA and inserted into EVs remain to be elucidated.

Similarly, it remains relatively unexplored whether any of the recently uncovered oncogenic mutations in adult and pediatric brain tumors manifest themselves, and contribute to the disease progression via the release of the related mutant proteins (or nucleic acids), as cargo of oncosomes. In this regard, some of the intriguing examples include IDH1 G395 mutations in high grade glioma (Yan et al., 2009), which have recently been detected in EV preparations from GBM culture medium and CSF (Balaj et al., 2012). Similarly, the expression of the gene fusion product KIAA1549/BRAF in juvenile pilocytic astrocytoma (JPA) (Jones et al., 2009) may be detectable in a similar manner, since another form of mutant BRAF (V600E) was found in EVs collected from plasma of melanoma patients (Ramachandran et al., 2011). Rapid progress in molecular characterization of adult and pediatric GBM has recently been extended to RTKs (PDGFR, MET) (Paugh et al., 2011), mutant forms of histone H3.3 and chromatin remodeling genes (Schwartzentruber et al., 2012), as well as several other events involving genetic and epigenetic alterations (Parsons et al., 2008; Lavon et al., 2010). It is noteworthy that by a simple analogy to the aforementioned studies on EGFRvIII, all of these newly discovered molecular changes may result in the emission of EVs endowed with signatures and biological activities resulting from their content of the respective mutant genes and gene products, and thereby may serve as biomarkers for molecular diagnosis of the underlying brain tumors (Al-Nedawi et al., 2009b).

The central nervous system (CNS) is also a site of non-neuroectodermal cancers including hematopoietic malignancies and hemangioma, as well as several secondary brain tumors with distinct molecular underpinnings. The ability of these tumors to elaborate and shed oncosomes still remains to be studied. Metastatic brain tumors often originate from distant cancers such as those of the lung, breast, skin (melanoma), and several other sites, and are associated with high morbidity, mortality, and therapeutic intractability. Virtually nothing is known about the role of oncosomes in such secondary brain tumors, in spite of the fact that these conditions have emerged as a growing therapeutic challenge (Steeg et al., 2011). Correlative studies resulted in detection of tumor-related DNA containing microsatellite markers of chromosome 3p alterations in plasma of patients with non-small cell lung cancer. This finding is indicative of a systemic disease, which often metastasizes to the brain (Lleonart et al., 1998). Also p53 sequences in plasma of ovarian cancer patients may segregate with a higher incidence of brain metastasis (Swisher et al., 2005). In all these cases the association between circulating DNA and the emission of tumor EVs is plausible but unproven. While GBM-derived EVs (Noerholm et al., 2012) and modified exosomes (Alvarez-Erviti et al., 2011) may cross (or circumvent) the blood brain barrier, this may not necessarily apply to the ability of naturally occurring extracranial EVs to access sites of brain metastases, and conversely, it is unknown whether EVs emanating from metastatic brain tumors can freely access systemic circulaton (Steeg et al., 2011). Therefore, it remains to be studied whether formation of metastatic niches in the brain is related to biological activities of cancer or stromal-derived EVs, whether oncosomes participate in these processes, and whether signatures of brain metastases can be found in patient plasma.

## The possible role of extracellular vesicles as biomarkers in brain tumors

Brain tumors represent a significant medical challenge due to their anatomical location, functional impact, and biological complexity. Primary brain tumors likely originate from different populations of NSCs and their major types include astrocytoma, oligodendroglioma, meningioma, ependymoma, and embryonal brain tumors such as MB, primitive neuroectodermal tumors (PNET), and atypical teratoid/rhabdoid tumor (AT/RT), each associated with different age-related incidence (Wrensch et al., 2002; Zhu and Parada, 2002; Stiles and Rowitch, 2008). Astrocytic brain tumors constitute the most prevalent and heterogenous brain tumor type in adults and are divided into grades I–IV, according to their histopathological and clinical characteristics. The most aggressive grade IV tumors are referred to as glioblastoma multiforme (GBM) and presently remain incurable (Wen and Kesari, 2008).

The rapid development of new technologies over the past two decades resulted in the recent explosion of profiling and sequencing studies that have profoundly altered the landscape of primary brain tumors (Li et al., 2012). Perhaps the most notable development in this regard is the subclassification of the traditional, clinically-based nosology into a multitude of molecularly distinct disease subtypes, each characterized by a distinct set of driver mutations, their related oncogenic pathways, and signature changes in the cellular transcriptome, proteome, miR-ome, and epigenome. This complexity carries enormous therapeutic implications as each molecular pathway of brain tumorigenesis and disease subtype may potentially require a different therapeutic paradigm, contain distinct molecular targets for therapy, and could be characterized by separate sets of diagnostic, prognostic, and predictive biomarkers.

Some of the more spectacular examples of recent developments in this regard include the large scale analysis of the mutational status of human GBM with extensive verification of several functional gains and losses (Parsons et al., 2008). In addition to the primary and secondary pathways of GBM progression involving some of the aforementioned genetic events [e.g., EGFR amplification and IDH1 mutation, respectively (Ohgaki and Kleihues, 2009)], high grade glioma is now recognized to consist of at least four major molecular subtypes (neural, proneural, classical, and mesenchymal), which differ in their genetic and epigenetic make-up (Verhaak et al., 2010). Although histologically similar, these tumors also differ from pediatric GBMs, which are characterized by distinct gene expression pattern and unique mutations, involving the growth factor RTKs (PDGFR or MET) (Paugh et al., 2011), mutant histones (H3.3) (Schwartzentruber et al., 2012), and several other genetic and epigenetic abnormalities. Similarly, in MB, several molecular subtypes have recently been discovered, and their molecular drivers (Myc, Wnt, SHH) described in some detail, along with unique genetic events that may separate primary and metastatic tumors in the same patient (Wu et al., 2012).

**Figure 3:**
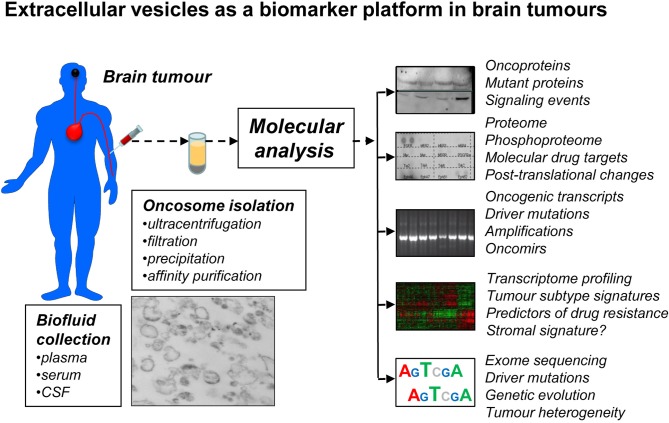
**Extracellular vesicles as a prospective biomarker platform in molecular diagnosis of primary and secondary brain tumors.** EVs that circulate in peripheral blood or cerebrospinal fluid (CSF) can be readily recovered using several existing methods and various microfluidic and nanotechnology platforms under development. It may be possible to enrich for tumor-derived EVs (oncosomes) using tumor markers (EGFRvIII) and/or specific immunoaffinity techniques. The cargo of oncosomes may be dissected for individual molecules (oncoproteins), their activation, posttranslational processing (phosphorylation) and combinations, including for the purpose of monitoring putative drug targets and their responses to targeted therapies. EVs may provide information as to new, or pre-existing mutations that may occur in brain tumor cells, as well as scope and phylogeny of driver events, for example by sequencing nucleic acids (DNA, RNA) in the cargo. Profiling of proteins and nucleic acids may reveal signatures of brain tumor subgroups and individual variations in gene expression. The challenge is to develop technologies that would ensure sensitivity, specificity, reproducibility and processing of this information for clinical purposes.

It is reasonable to predict that, as in the case of EGFRvIII in adult GBM, many (if not all) of these driver, passenger, and signature mutations, in high and low grade adult and pediatric glioma, MB, ependymoma, and other tumors may be present in the corresponding patient plasma as cargo of EVs. Should the appropriate detection methods be developed, EV platform could become invaluable for early diagnosis, subtype determination, longitudinal monitoring of the disease progression, and adaptive following of therapeutic responses (Figure 3). These are but a few examples that illustrate the evolving oncogenic landscape of human brain tumors and translational opportunities that EV emission may present in this context.

In addition to the unexpectedly complex molecular nature of brain tumors, they also exhibit considerable intra-lesional heterogeneity. Recent evidence suggests that while certain (classical subgroup) GBM lesions contain regions positive for EGFRvIII expression, this signal may be consistently absent in adjacent tumor tissues (Biernat et al., 2004). Experimental evidence suggests that such oncogenic mosaic is maintained in an active manner by paracrine interactions between cancer cell populations, a process that involves interleukin 6 and other mediators (Inda et al., 2010). Moreover, different regions of GBM may contain clones expressing different oncogenic mutations of cellular RTKs, such as amplified EGFR or PDGFR (or their mixture) (Snuderl et al., 2011). Temporal changes in molecular profiles of brain tumors have also been detected. This spatiotemporal and regional complexity may result in significant sampling errors and diagnostic challenges, especially when coupled with limited and highly invasive access to brain tumor tissue (through surgery or biopsy).

In this regard, the ability of brain tumor cells to shed EVs containing oncogenic, mutant, or otherwise cancer-specific molecular cargo opens a new window of opportunity (Figure 3). As we and others have suggested earlier, the access to EVs circulating in blood or CSF may provide an unprecedented glimpse into the repertoire of molecular alterations occurring in individual cancer patients and in real time. As mentioned previously, EVs isolated from plasma of mice harboring GBM xenografts (Al-Nedawi et al., 2008) and from GBM patients (Skog et al., 2008) have already provided a proof of principle in this regard (e.g., detection of EGFRvIII). Preliminary experiments with mouse tumors suggest that this approach may not only permit the analysis of mutant DNA or RNA (and their variations), but also detection of proteins that serve as targets for biological therapeutics (e.g., phospho-EGFR). The related molecular responses may also be reflected in circulating EVs (e.g., EGFR dephosphorylation) (Al-Nedawi et al., 2010). In a few anecdotal cases of GBM, plasma EV samples were found to be positive for the mutant EGFRvIII, while this signal was weak or undetectable in the corresponding surgical tumor tissue (Skog et al., 2008; Al-Nedawi et al., 2010). This may suggest that in those rare cases the EGFRvIII expressing regions of the tumor may have been missed during tissue collection, but the presence of this oncogene could be captured by the analysis of EVs.

The same principle could be extrapolated to tumor-associated stromal cells (microglia, astrocytes, vascular, and inflammatory cells), many of which possess the ability to vesiculate, and their EVs may reveal their functional and molecular states. Since molecular profiling of tumor-associated host cells (*in situ*) has already translated into valuable prognostic information in breast cancer (Finak et al., 2008), so too, at least hypothetically, could the analysis of stromal cells and their related circulating EVs in primary and secondary brain tumors.

While considerable technological barriers still do exist, the analysis of EVs (“vesiculome”) in patients with brain tumors may provide unique information as to oncogenic driver mutations, molecular signatures, oncogenic pathways, disease subgroups, drug-resistance-associated mutations (e.g., mutations of EGFR), and other markers. Similarly, temporal changes in the molecular make-up of the tumor could potentially be monitored in individual patients, along with the magnitude of drug responses. In addition the evidence of stem cell markers, microenvironmental responses (e.g., by measuring levels of hypoxia-regulators such as HIF or CAIX) and many other parameters could conceivably be extracted from the molecular cargo of circulating EVs (Figure 3). If successful, these approaches could have a major impact on the design of biomarker-driven clinical trials, drug development, and ultimately the outcomes in brain tumors.

## Summary

Cellular and regional heterogeneity as well as intercellular communication emerge as key elements in the pathogenesis of brain tumors. In this regard, the involvement of EV-mediated molecular trafficking represents an intriguing aspect, especially as it relates to the horizontal transfer of molecular triggers of cellular transformation: oncogenes, tumor suppressors, and mediators of genetic instability (Rak and Guha, 2012). In so doing, EVs could reprogram cellular phenotypes and recruit indolent and normal cells to participate in angiogenesis, invasion, dissemination, and other events. It is conceivable that a limited numbers of cancer cells that underwent the initial mutation may generate a larger “oncogenic field” by emitting EVs harboring mutant genes (Figure 2). It is possible, therefore, that the intercellular trafficking of EVs may serve as a target for new anticancer therapeutics (Al-Nedawi et al., 2009a).

While the relative contribution of EV trafficking to the biology of different types of primary and metastatic brain tumors remains to be thoroughly investigated, the emission of EVs containing molecular signatures may offer unprecedented diagnostic opportunities. Development of new technologies (including microfluidics and nanotechnology) that would secure a non-invasive, remote, and repeated access to biological information encapsulated in circulating EVs has already begun (Shao et al., 2012). Thus, a better understanding of the link between cellular transformation and vesiculation processes may have an enabling influence on the future progress in individualized patient care.

### Conflict of interest statement

The authors declare that the research was conducted in the absence of any commercial or financial relationships that could be construed as a potential conflict of interest.
